# Budding-like division of all-aqueous emulsion droplets modulated by networks of protein nanofibrils

**DOI:** 10.1038/s41467-018-04510-3

**Published:** 2018-05-29

**Authors:** Yang Song, Thomas C. T. Michaels, Qingming Ma, Zhou Liu, Hao Yuan, Shuichi Takayama, Tuomas P. J. Knowles, Ho Cheung Shum

**Affiliations:** 1 Department of Mechanical Engineering, The University of Hong Kong, Pokfulam, 999077 Hong Kong; 2 Wallace H. Coulter Department of Biomedical Engineering, Georgia Institute of Technology, Atlanta, GA 30332 USA; 3 Department of Chemistry, University of Cambridge, Lensfield Road, Cambridge, CB2 1EW UK; 4 Paulson School of Engineering and Applied Sciences, Harvard University, Cambridge, MA 02138 USA; 5 HKU-Shenzhen Institute of Research and Innovation (HKU-SIRI), 518000 Shenzhen, China; 6 Cavendish Laboratory, Department of Physics, University of Cambridge, JJ Thomson Avenue, Cambridge, CB3 0HE UK

## Abstract

Networks of natural protein nanofibrils, such as cytoskeletal filaments, control the shape and the division of cells, yet mimicking this functionality in a synthetic setting has proved challenging. Here, we demonstrate that artificial networks of protein nanofibrils can induce controlled deformation and division of all-aqueous emulsion droplets with budding-like morphologies. We show that this process is driven by the difference in the immersional wetting energy of the nanofibril network, and that both the size and the number of the daughter droplets formed during division can be controlled by modulating the fibril concentration and the chemical properties of the fibril network. Our results demonstrate a route for achieving biomimetic division with synthetic self-assembling fibrils and offer an engineered approach to regulate the morphology of protein gels.

## Abstract (web summary)

The cytoskeleton, a network of fibrils, controls how cells divide. Here, the authors show that synthetic protein fibrils added to an emulsion can control the division of droplets and that this method can be used to control the morphology of microparticles during biomaterial preparation.

## Introduction

The growth of liquid protrusions from soft biological interfaces, including membranes, is a process which underlies many cellular processes, such as asexual reproduction of yeast cells^1^, active transport of macromolecules through endocytosis^2^, as well as the blebbing during the programmed cell death^3^. From a thermodynamic point of view, the formation of budding protrusions and their subsequent fission into daughter droplets is an energetically unfavorable processes^4, 5^ being associated with an increase of interfacial area. The formation of surface protrusions in liquid droplet systems^6–9^, therefore, requires interfacial engineering by means such as incorporating proteins into the membrane surrounding the droplet^6, 7^, inducing the dewetting of sub-droplets from vesicles with multi-phase compartments^8, 9^, or activating chemical reactions that destabilize the droplet interface^10, 11^. The formation of such membrane protrusions in response to environmental stimuli^12^ can lead to the complete fission of the daughter droplets.

Living cells are able to control their shape and division using networks of protein nanofibrils, such as the cytoskeleton^13–16^. Network-mediated cellular division, such as the condensation of the septal Z-ring^17–19^ in dividing bacteria, and the polymerization (or depolymerization) of actin filaments in eukaryotic cells^20, 21^ are all related to the functioning of the protein networks at variable fibril concentrations. However, mimicking natural fibril-network-mediated division remains challenging, even though this functionality could have significant applications in a synthetic setting. Synthetic cytoplasmic matrices could provide a bottom-up approach^22^ to unravel the role of protein networks in the division of protocells. Water-in-water (w/w) emulsion droplets, formed for instance by dispensing a dextran-rich aqueous phase into an immiscible polyethylene glycol (PEG)-rich continuous aqueous phase, have been used previously to simulate compartmentalized cytoplasm^23, 24^. All-aqueous emulsions are particularly advantageous in this context, due to the characteristic ultra-low interfacial tension^25^ (<1 × 10^−3^ N m^−1^) which dramatically lowers the energetic cost for interfacial area increase during droplet division.

In this paper, we demonstrate that the addition of protein nanofibrils to all-aqueous emulsions can induce the division of the w/w emulsion droplets and that the concentration of fibrils controls the division regimes of budding droplets. Our observations not only provide a simplified physical model for reproducing droplet division in a synthetic setting, but also inspire engineered approaches to adjust the surface morphology of protein gels.

## Results

### Gelation of protein nanofibril suspensions

Protein nanofibrils were synthesized by polymerizing lysozyme monomers at 65 °C under acidic conditions (pH = 1.6, see Methods)^26^. After cooling to room temperature, the nanofibril suspension (2 wt%) formed a soft gel. By introducing shear forces through stirring, the nanofibril gel transformed into a viscoelastic fluid (see Supplementary Fig. 1), but returned to the gel phase under quiescent conditions. The gelation of the fibril suspension could be controlled by dissolving additional solutes in the aqueous medium. For example, when the fibril suspension was injected slowly into a 10 wt% dextran solution, it formed a gel (see Supplementary Fig. 1d). However, when injected into a 8 wt% PEG solution, the fibrils remained suspended in solution without undergoing gelation, probably due to the incorporation of PEG molecules into the fibril network.

### Division of w/w drops loaded with protein nanofibrils

**Fig. 1:**
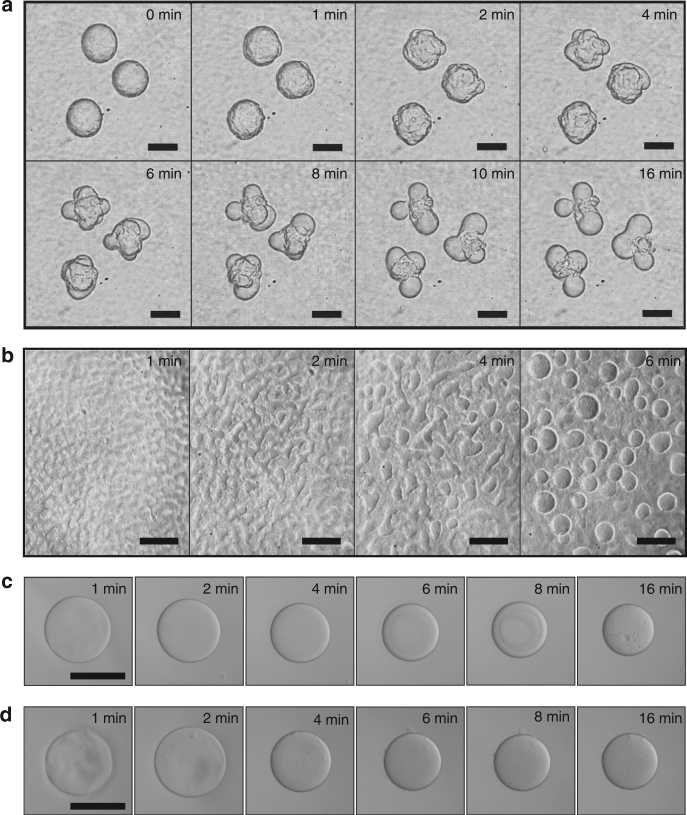
Budding-like division of w/w droplets loaded with protein nanofibrils. **a** Optical microscope images of dividing w/w emulsion droplets preloaded with protein nanofibrils. The droplet phase was preloaded with 7.5 wt% dextran T500 and 1.2 wt% lysozyme nanofibrils before injection into a 8 wt% PEG-10 mM HCl solution. **b** Optical microscope images showing the formation of protrusions on a flat w/w interface. No splitting of w/w droplets was observed when the concentration of fibrils was **c** 0 wt% and **d** 0.1 wt%. Scale bars are 200 μm

An aqueous suspension of 1.2 wt% fibrils in 7.5 wt% dextran T500 was dispersed into an acidic PEG (8 wt%, Mw = 20,000, pH = 3) solution via electrospray^27^, resulting in the formation of dextran-in-PEG w/w emulsion droplets. Due to the higher osmolality of the PEG-rich continuous phase, the droplets underwent dehydration until a balance was established between the osmolality of the dextran-rich phase and that of the PEG-rich phase. During droplet shrinking, small buds were observed to form on the droplet surface (Fig. 1a). The diameter of these buds increased over time due to coalescence; eventually, each mother droplet split into a well-defined number of daughter droplets. Similar protrusions were observed to form also on flat w/w interfaces (Fig. 1b). The formation of buds was strongly dependent on the presence of nanofibrils in the dextran-rich droplet phase: no protrusions were observed under the same conditions of osmotic pressure and w/w interfacial tension without loading a sufficient amount of fibrils into the droplet phase (Fig. 1c, d).

### Mechanism of budding-like division of w/w droplets

**Fig. 2:**
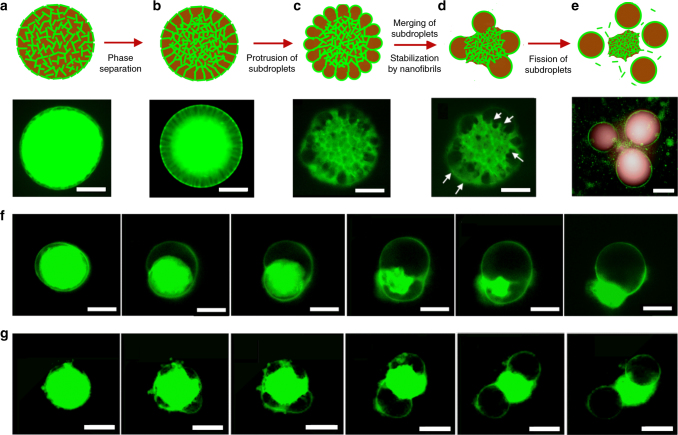
Mechanism of budding-like division of w/w emulsion droplets mediated by protein nanofibrils. **a**–**e** Schematic diagram and fluorescence microscope images describing the mechanistic steps in the budding-like division of w/w droplets. The fibril network (stained green) contracts and phase-separates from the remaining liquid phase through a dewetting transition. In this transition, the as-formed protrusions coalesce (as pinpointed by the white arrows) until a sufficient amount of fibrils adsorbs at the w/w interface to stabilize daughter droplets. Complete fission of dextran-rich subdroplets (faked red color) is observed after total decomposition of the fibril networks in the PEG-rich continuous phase. Scale bars, 100 μm. Fluorescence microscope images showing **f** single division (*C*_fibril_ = 0.5 wt%) and **g** multiple division (*C*_fibril_ = 1.0 wt%) of w/w droplets preloaded with protein nanofibrils and 8% dextran. Scale bars, 200 μm. The continuous phase consists of 8 wt% PEG dissolved in 10 mM HCl

To probe the mechanism behind the observed budding-like division of w/w emulsion droplets, we labeled protein nanofibrils with the fluorescent dye Thioflavin T (ThT)^28^. After homogenization of the ThT-labeled fibril suspension in the dextran phase, the droplet phase was immediately electrosprayed onto the continuous phase to form w/w emulsion droplets. Water was partially extracted from the droplet phase due to a prevailing osmotic pressure (30 mOsm kg^−1^) between the two immiscible aqueous phases. Since fibrils do not permeate the w/w interface, droplet dehydration resulted into an increase of fibril concentration in the droplet phase. Above a critical fibril concentration, a viscoelastic fibril network composed of fibril bundles could be seen to contract in response to an abrupt change in osmotic pressure and then phase-separate from the dextran-rich droplet phase (see Fig. 2a, b and Supplementary Fig. 2). The phase separation originates from the intrinsic incompatibility of the fibril network with the dextran-rich phase and the consequent need to reduce the interfacial area between the two phases. The contraction of viscoelastic fibril network pulls the droplet interface, leading to buckled interfaces (Fig. 2c). After phase separation, the fibril network underwent spontaneous dewetting from complete to partial wetting of the network in the droplet phase (Fig. 2d, e); the interface between the network and the dextran-rich phase is indeed decreased during budding, as seen in Fig. 2f and g. The dewetting transition is driven by (see Supplementary Note 1):

1 $$\sigma _{{\mathrm{PEG/net}}} - (\sigma _{{\mathrm{dex/net}}} + \sigma _{{\mathrm{w}}/{\mathrm{w}}}\cos \theta ) < 0,$$

where $\sigma _{{\mathrm{w}}/{\mathrm{w}}}$ is the w/w interfacial tension, and $\sigma _{{\mathrm{dex}}/{\mathrm{net}}}$ and $\sigma _{{\mathrm{PEG}}/{\mathrm{net}}}$ are, respectively, the interfacial tensions between the fibril network and the dextran-rich and PEG-rich phases; $\theta$ is the wetting angle formed by the dextran-rich phase and the fibril network. The driving force for dewetting is favorable when the expression in Eq. (1) is negative. This condition is satisfied in our experiments because $\sigma _{{\mathrm{PEG/net}}} \approx 0$ (the fibril network does not phase separate in the PEG-rich phase); moreover, under all the tested experimental conditions, we measured $\sigma _{{\mathrm{dex}}/{\mathrm{net}}} < \sigma _{{\mathrm{w}}/{\mathrm{w}}}$ (see Supplementary Fig. 3), leading to a final wetting angle of $\theta = \arccos ( - \sigma _{{\mathrm{dex}}/{\mathrm{net}}}/\sigma _{{\mathrm{w}}/{\mathrm{w}}}) > 90^\circ ,$ in agreement with the observations of Fig. 2f and g. Such a large wetting angle is consistent with the higher partitioning affinity of fibrils to the PEG-rich phase compared to the dextran-rich phase (Supplementary Fig. 4)^29^.

**Fig. 3:**
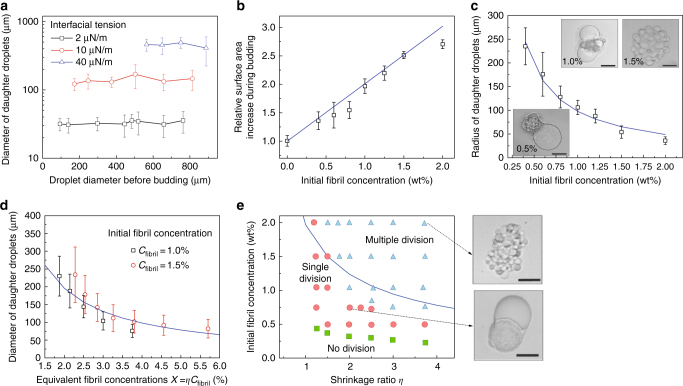
Fibril networks control the number and size of daughter droplets. **a** The average diameter of daughter droplets is independent of the size of the mother droplet but increases linearly with increasing w/w interfacial tension. **b** After decomposition of fibril networks, the total surface area of the daughter droplets increases linearly with initial fibril concentration. **c** Radius and number of stabilized protrusions as a function of fibril concentration. Scale bars, 100 µm. **d** Diameter of stabilized daughter droplets as a function of the product of shrinkage ratio (*η*) and initial fibril concentration (*C*_fibril_). **e** Phase diagram summarizing the effects of shrinkage ratio and initial fibril concentration on the division regimes of w/w droplets loaded with protein nanofibrils. The initial diameter of the mother droplet was 250 ± 20 µm. Inset: optical microscope images showing representative w/w droplets in the multiple (top) and single (bottom) division regimes. Scale bars are 200 µm. The solid blue lines in **a**–**e** are predictions from Eq. (2). The size of daughter droplets, measured from 50 replicates, are expressed as (mean ± s.d.)

As the viscoelastic fibril network contracts under the osmotic pressure (see Supplementary Fig. 5 and Supplementary Note 2), the dextran-rich phase is squirted out of the porous fibril network, resulting in the formation of liquid protrusions with large interfacial area, in analogy to what happens when a water-absorbing sponge is squeezed in oil. In the absence of any stabilizing mechanism, the protruded subdroplets are unstable against coalescence and their average radius, *R*, increases over time. The merging of the daughter droplets, however, can be halted by the adsorption of fibrils at the w/w interface. We have previously reported that protein nanofibrils efficiently stabilize dextran-in-PEG emulsion droplets due to the entrapment of fibrils at the w/w interface^29^. If the total amount of adsorbed fibrils is insufficient to fully cover the w/w interface, the surface protrusions coalesce in order to minimize the total interfacial area, $A \propto \frac{1}{R}$. However, as the daughter droplets coalesce, the w/w surface coverage by fibrils progressively increases in proportion to *R* until droplet coalescence is eventually halted. In analogy to the physics of Pickering emulsions^30^, the average diameter *D* = 2*R* of daughter droplets stabilized by adsorbed protein fibrils can be estimated as a function of the initial concentration of fibrils in the mother droplet, *C*_fibril_, as (see Supplementary Note 3 and Supplementary Fig. 6):

2 $$D_{{\mathrm{daughter}}}{\mathrm{ = }}2R_{{\mathrm{daughter}}}{\mathrm{ = }}\frac{{3a\sigma _{{\mathrm{w/w}}}}}{{(\sigma _{{\mathrm{dex/net}}}{\mathrm{ - }}\sigma _{{\mathrm{PEG/net}}})\eta C_{{\mathrm{fibril}}}}},$$

where $a$ is the radius of the cross-section of fibril bundles, and *η* is the shrinkage ratio, i.e., the ratio between the initial volume of the mother droplet and the total volume of daughter droplets. Equation (2) predicts that the diameter of daughter droplets, *D*_daughter_, increases in proportion to $\sigma _{{\mathrm{w}}/{\mathrm{w}}}$ (see Fig. 3a, Supplementary Fig. 7, Supplementary Note 4, and Supplementary Table 1) and decreases in proportion to the fibril concentration, *C*_fibril_, and the shrinkage ratio *η*. Interestingly, *D*_daughter_ is not affected by variations in the volume of the mother droplets (see Fig. 3a and Supplementary Fig. 8).

### Effect of fibril concentration on droplet division

According to Eq. (2), the initial concentration of fibrils in the droplet phase, *C*_fibril_, controls the final size of the daughter droplets by determining the availability of excess fibrils that can stabilize the w/w interface. To test this prediction, we varied systematically the concentration of fibrils in the droplet phase while leaving the other parameters unchanged. When the fibril concentration was below 0.2 wt%, no phase separation into a fibril network was observed (Fig. 1c, d) and, as a result, droplets remained stable over time without undergoing division. When the fibril concentration was above 0.3 wt%, a fibril network was observed to phase-separate from the dextran-rich phase after droplet dehydration. Under these conditions, however, the amount of fibrils was insufficient to stabilize the surface protrusions; as a result, the daughter droplets were observed to merge into a single droplet before total decomposition of the fibril network into the PEG-rich phase (single-division regime, see Fig. 2f). When the concentration of fibrils was above a critical value $C_{{\mathrm{fibril}}}^ \ast$ (1.0 wt%), the formation of multiple stable protrusions was observed (multiple-division regime, Fig. 2g). In this regime, the total interfacial area of the daughter droplets was significantly larger than that of the mother droplet^31^. The relative increase in w/w interfacial area was found to scale linearly with initial fibril concentration (see Fig. 3b and Supplementary Note 5). Furthermore, the radius of the stabilized surface protrusions was found to decrease with increasing fibril concentration (Fig. 3c), in agreement with our theoretical predictions. Finally, we measured the size distribution of dividing droplets (see Supplementary Figs. 9, 10) and found that, due to the enhanced stabilization of daughter droplets, higher fibril concentrations were associated with a higher degree of size monodispersity.

### Effect of shrinkage ratio on droplet division

As a next step, we investigated the role of shrinkage ratio *η* on droplet division. To do so, we reduced the concentration of dextran in the droplet phase from 13 to 4 wt%, while maintaining the initial fibril concentration and the PEG concentration in the continuous phase constant. With a lower concentration of dextran in the droplet phase, a higher osmotic pressure is created to extract water from the droplet and induce budding (Supplementary Fig. 11). This resulted in a higher degree of dehydration and, consequently, a denser fibril network. The average diameter of the daughter droplets was found to decrease with increasing shrinkage ratio *η* (Fig. 3d), in agreement with the prediction of Eq. (2). The experiment was performed for two different fibril concentrations. Combining the effect of shrinkage ratio, *η*, and initial fibril concentration, *C*_fibril_, we define a combined parameter *Χ* = *ηC*_fibril_ that reflects the fibril concentration after dehydration of the mother droplet. The average radius of the daughter droplets was found to decrease inversely proportional to this combined parameter *Χ*, confirming that the final diameter of the stabilized dextran-rich buds can be equally modulated by changing either the initial fibril concentration or the droplet shrinkage ratio (Fig. 3d, e and Supplementary Note 6).

### Fabrication of protein microgels with surface protrusions

**Fig. 4:**
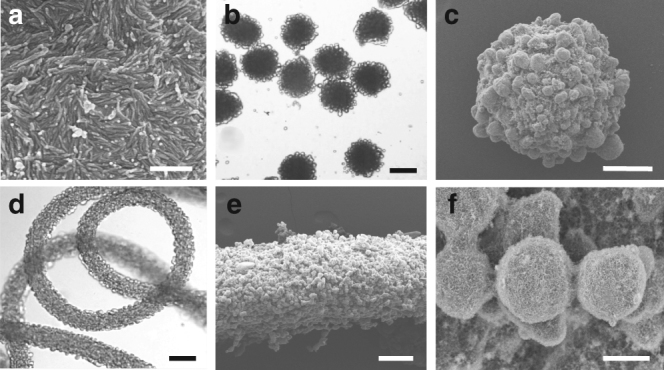
Microfluidic fabrication of protein microparticles and microfibers with surface protrusions. **a** Scanning electron microscope (SEM) image of fibril networks. Scale bar, 500 nm. **b** Optical microscope and **c** SEM images of microbeads with surface protrusions. **d** Optical microscope and **e** SEM images of a microfiber with **f** surface protrusions. Scale bars: 200 μm (**b, d**), 100 μm (**c**, **e**), and 10 μm (**f**)

The budding-like division of w/w emulsion droplets using protein nanofibrils demonstrated in this paper can be applied to induce surface protrusions in protein hydrogels. We have previously reported on a microfluidic approach to generate all-aqueous emulsions and jets^32^. By incorporating fibril networks into the dispensing phase, we could induce small protrusions on the surface of the droplets and jets. The dextran-rich buds could be solidified by means of osmotic dehydration in the continuous PEG phase (>20%), and the speed of dehydration increased with the PEG concentration (see Methods^33^). By balancing the speed of dehydration and the budding kinetics, the dextran-rich protrusions could be immobilized on the surface of the microparticles and microfibers, as shown by the optical and scanning electron microscope (SEM) images in Fig. 4. The physical dimensions of the microparticles and fibers as well as the sizes of the formed surface protrusions can be tuned by adjusting the osmotic pressure or the fibril concentration in the dispersed phase. This approach allows control of surface roughness^34, 35^ and design of surface patches^36^ of protein-based hydrogels in the all-aqueous environment, which will be of interest for the micropatterning of biomolecules and cells^37^ on the surface of biomaterials, such as cell-laden matrices, protein-delivery vehicles, and tissue engineering scaffolds^38^.

In this study we have suggested a possible route for achieving division of liquid droplets by exploiting synthetic protein fibrillization. Based on these results, we have further developed an all-aqueous platform for controlling the morphology of protein-rich gels, which could have important implications in tissue engineering and cell-mimicking studies.

## Methods

### Fabrication of fibril networks

An aqueous solution of chicken egg white was prepared by dissolving 2 wt% of the protein into a solution of 206 mM hydrochloric acid (HCl, Aladdin Trade Co. Ltd) containing 2 mM sodium chloride (NaCl, Sigma-Aldrich). The solution was incubated at 65 °C for 70 h to induce polymerization of protein monomers into long fibrils. To achieve complete conversion from monomers to mature fibrils, the colloidal suspension was stirred at a centrifugal speed of 7 × *g*. The fibril suspension was diluted to 0.25–2 wt% by adding water. The dynamic viscosity of the suspension was measured using a microfluidic viscometer (μVISC, Rheosense, Inc.).

### Fabrication of w/w droplets

The emulsion phase was prepared by vortex mixing dextran T500 (Shanghai Ryon Biological Co., Ltd.) with the fibril suspension (see fabrication of fibril networks). The final concentration of dextran in the emulsion phase was 5–12 wt%. The continuous phase was prepared by dissolving 8 wt% PEG (Mw = 20,000, Aladdin Trade Co., Ltd.) in an aqueous solution of 10 mM HCl.

W/w emulsion droplets were generated by using an all-aqueous electrospray setup^27^. The emulsion phase was charged positively (~2.5 kV), and subsequently sprayed into the continuous phase through a glass capillary with a nozzle diameter of 80 μm. A negatively charged metallic ring, with an enclosed circle of 4 cm in diameter, was positioned 1 mm beneath the capillary nozzle. The flow rate of the dispersed phase was maintained at 0.5 mL h^−1^.

### Microscopy observation

The process of budding was monitored by using fluorescence microscopy (DMIL, LED, Fluo Leica): the dextran-rich phase was tagged by dissolving 0.3 wt% FITC-DEAE-Dextran (Mw = 70,000, Life Technology Co., Ltd.) into the droplet phase. In separate trials, the networks of lysozyme fibrils were stained by 15 μM L^−1^ ThT (Shanghai Ryon Biological Co., Ltd.), followed by incubation of the resultant mixture at 60 °C for 15 min. The fluorescence staining was excited by using a blue laser source and the images were captured in the green channels. We also obtained corresponding bright-field (or phase-contrast) images of the budding droplets for measuring the volume of the w/w droplets.

### Osmolarity and interfacial tension measurements

Osmolarity of the emulsion phases before and after budding were measured using an osmometer (Model 3320, Advanced Instrument, Inc., USA). To determine the osmotic pressure between the droplet and continuous phases, we measured the osmolarity of dextran solutions before and after dehydration in the PEG-rich phase without addition of lysozyme fibrils, because the contribution of fibril networks to the osmolarity change of the emulsion phase can be ignored. After the splitting of droplets, the equilibrium concentration of dextran was determined from the phase diagram of dextran-PEG-H_2_O reported in ref. ^39^. The interfacial tension between different aqueous phases was measured using a spinning drop tensiometer (Krüss, Site 100). The protocol of measurement is illustrated in ref. ^25^.

### Fabrication of microparticles

An aqueous phase containing 2 wt% fibrils and 10% dextran T500 (droplet phase) was electrosprayed into the continuous phase consisting of an aqueous mixture of 25 wt% PEG (Mw = 2000, Aladdin Trade Co., Ltd.) and 0.1 wt% fibrils. At such a high concentration of PEG in the continuous phase, water was gradually extracted out of the droplet and the dextran-rich phase was solidified into solid particles. Glutaraldehyde (2.5 wt%, Sigma) was added into the continuous phase to cross-link the protein nanofibrils. After reacting for 12 h at 37 °C, the microparticles with budding surfaces were formed.

### Fabrication of microfibers

The above emulsion and continuous phases were separately injected through a co-flowing capillary microfluidic device, forming a stable dextran-in-PEG w/w jet. The flow rates of the dispersed and the outer continuous phases were maintained at 0.5 mL h^−1^ and 2 mL h^−1^, respectively. Subsequently, the w/w jet was collected in a rotating culture dish (Φ = 8 cm, 10 rpm, ~0.004 × *g*). The culture dish contained a mixture solution of 30 wt% PEG (Mw = 2000) and 2.5 wt% glutaraldehyde (Sigma). The w/w jet solidified into microfibers after incubation at 37 °C for 4 h.

### Scanning electron microscopy

Microparticles and fibers were transferred into absolute ethanol to remove the remaining water. The particles and fibers were then immersed into liquid carbon dioxide and dried following the protocols of critical point drying^40^. The dried samples were sputtered with gold before imaging under scanning electron microscopy (Hitachi S4800 FEG, 5 kV).

### Data availability

The authors declare that the data supporting the findings of this study are available within the paper and its Supplementary Information files and from the authors upon reasonable request.

## Electronic supplementary material

Supplementary Information
