# Supplementary material for: Budding-like division of all-aqueous emulsion droplets modulated by networks of protein nanofibrils
Source: Nat Commun. 2018 May 29;9:2110. doi: 10.1038/s41467-018-04510-3 (PMC5974351; doi:10.1038/s41467-018-04510-3)
Supplement: Supplementary file 1 — Supplementary Information [file 41467_2018_4510_MOESM1_ESM.pdf]

Supplementary Information

Budding-like Division of All-Aqueous Emulsion Droplets Modulated by Networks of Protein Nanofibrils

by Song et al.

Correspondence should be addressed Dr. Ho Cheung Shum. Email: ashum@hku.hk

Supplementary Figures

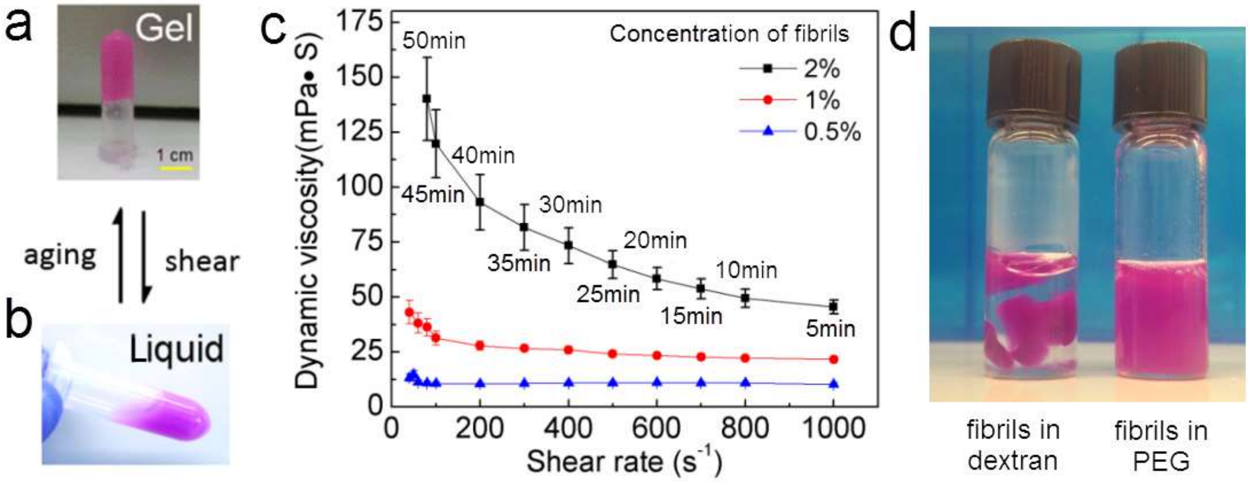

Supplementary Fig. 1 Gel-fluid transition and the viscoelasticity of amyloid nanofibril networks. (a) The fibril suspension (2 wt%) forms a gel after incubation for 1-2 hours. (b) Shaking the gel yields a liquid suspension. (c) A plot of the dynamic viscosity of the viscoelastic fibril network suspension at different fibril concentrations as a function of shear rate. The shear rate is lowered stepwise from 1000 s^{-1} to a near-zero shear rate in intervals of 5 minutes. Error bars represent standard deviation; each measurement was taken with 3 replicates. (d) When a 2 wt% fibrils suspension is slowly injected into a 10% dextran solution, the fibril network remains a solid gel; on the other side, when the 2 wt% fibril suspension is slowly injected into a 8% PEG solution, a suspension of dispersed fibrils is obtained. Fibrils are stained by Nile red.

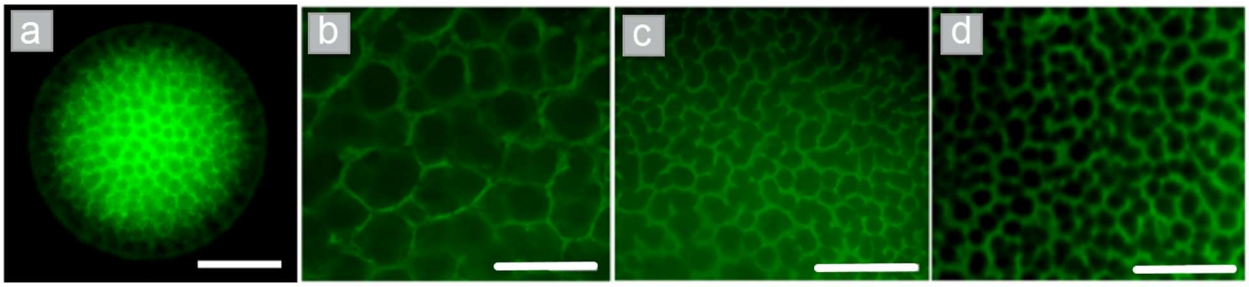

Supplementary Fig. 2 Formation of amyloid fibril network in the dextran-rich droplet phase. (a) Fluorescence microscope images showing the formation of fibril meshes. The fibril mesh is composed of fibril bundles. Scale bar is 100 µm. (b)-(d) As the fibril concentration increases, the mesh size of the fibril network decreases. The fibril concentrations are (b) 0.5 wt%, (c) 1.2 wt% and (d) 2.0 wt%, respectively. Scale bars are 50 µm. The average width of the fibril bundles, is 1-2 µm, and does not vary significantly with the concentration of fibrils preloaded in the mother droplet.

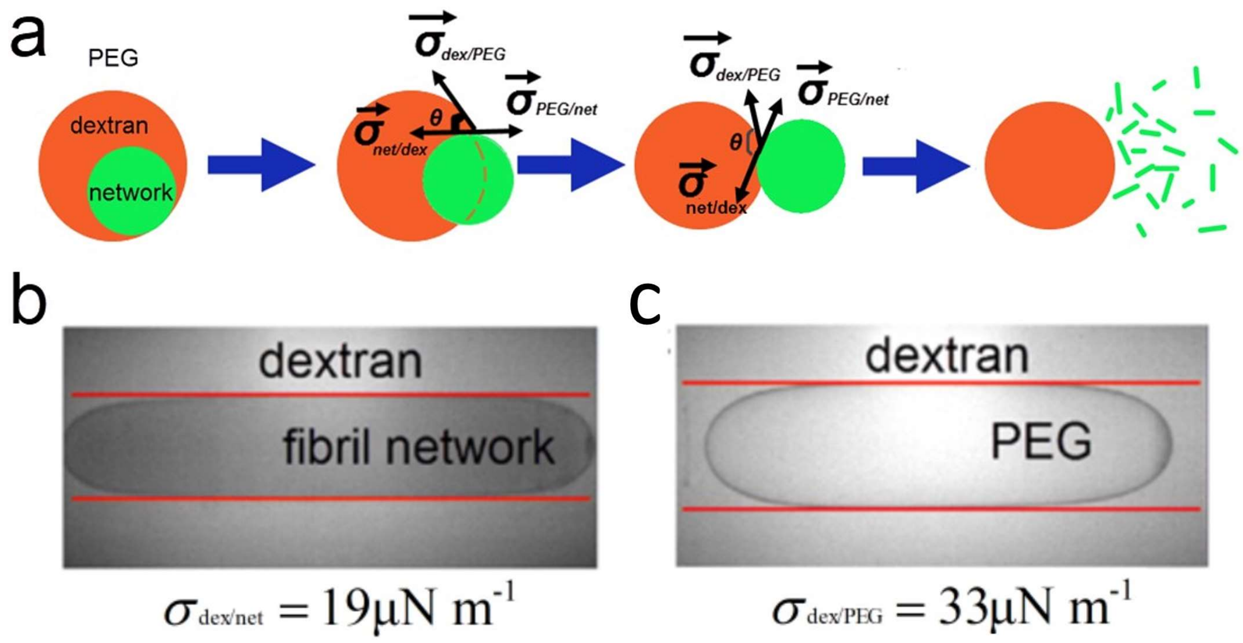

Supplementary Fig.3 Interfacial tensions driven dewetting of fibril networks from droplets. (a) A schematic diagram showing the dewetting of the dextran-rich droplet from the fibril networks. (b, c) Measurement on interfacial tensions between immiscible aqueous phases using a tensiometer. (b) The inner phase contains 2% fibrils and the outer phase is 15% dextran solution with 10mM HCl. The measured interfacial tension between the dextran-rich phase and the fibril network phase is 19 $\mu N\ m^{-1}$. (c) The inner phase contains 8% PEG and the outer phase is 15% dextran solution with 10mM HCl. The measured interfacial tension between the dextran-rich and the PEG-rich phase is 33 $\mu N\ m^{-1}$.

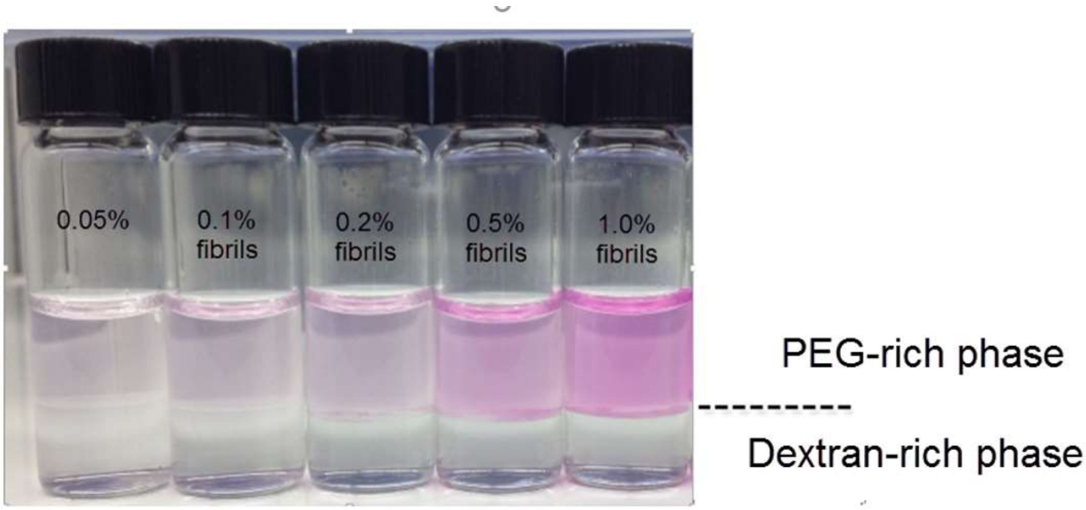

Supplementary Fig. 4 Lysozyme fibrils have a higher partitioning affinity to the PEG-rich phase than to the dextran-rich phase. Fibrils are stained with Nile red. Because of their density difference, the PEG-rich phase floats on the top of the dextran-rich phase. The higher affinity of fibrils to the PEG-rich phase enables selective stabilization of dextran-in-PEG emulsion (see Supplementary Reference 1).

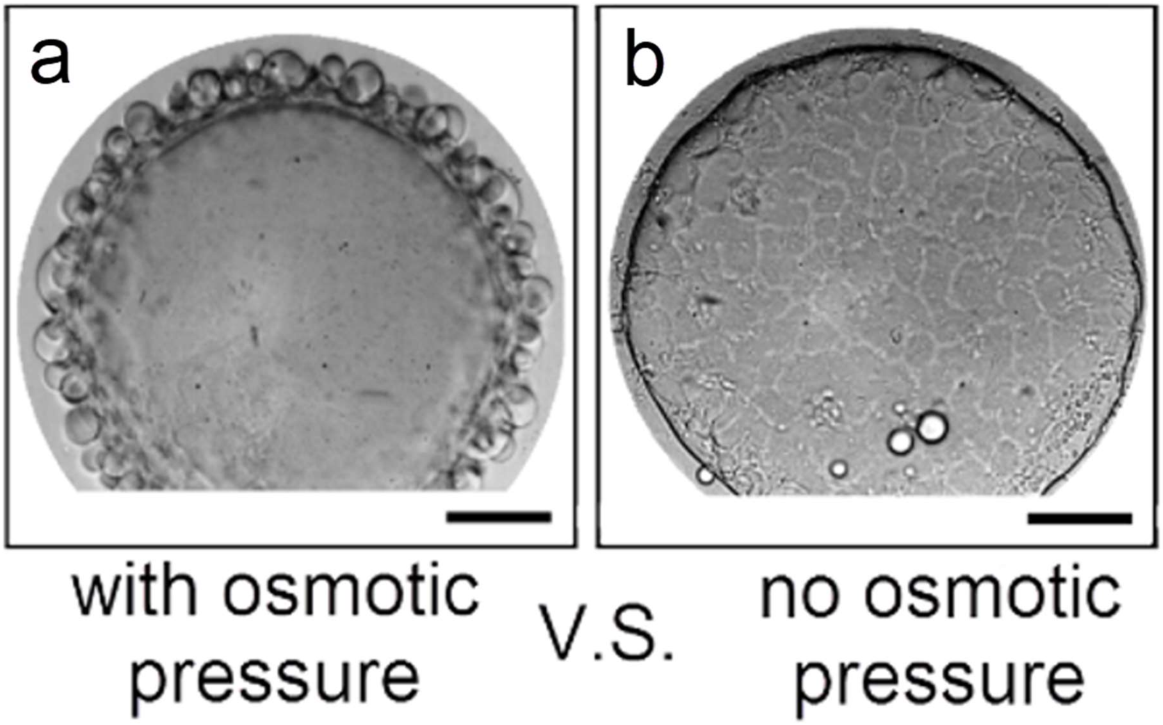

Supplementary Fig. 5 Presence of Osmotic pressure is required to induce the protrusion of the buds. (a) Droplet buds can grow when an osmotic pressure (40mOsm Kg^{-1}) exists between the dextran and PEG phases. The concentration of fibrils in the droplet phase is 2%. (b) When no osmotic pressure is applied, no buds protrude from the surface of the mother droplet. Instead, only some flat patches are observed on the surface of the mother droplet under the optical microscope. Scale bars are 100 μm.

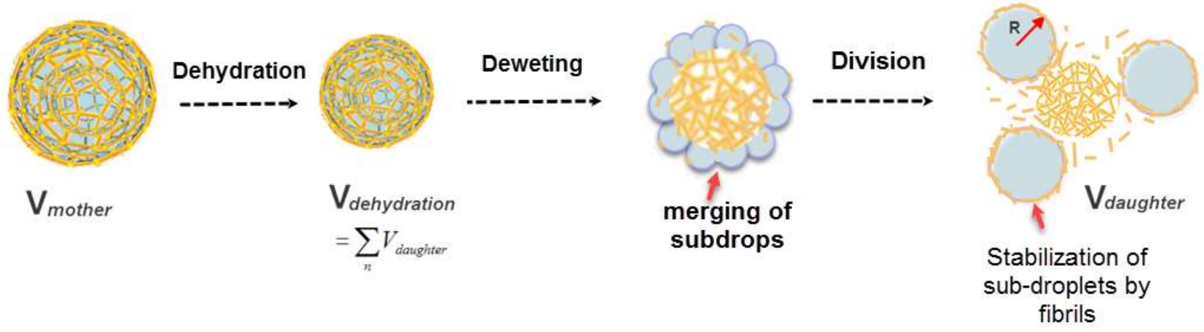

Supplementary Fig. 6 A simplified scheme of the droplet division mediated by protein nanofibrils.

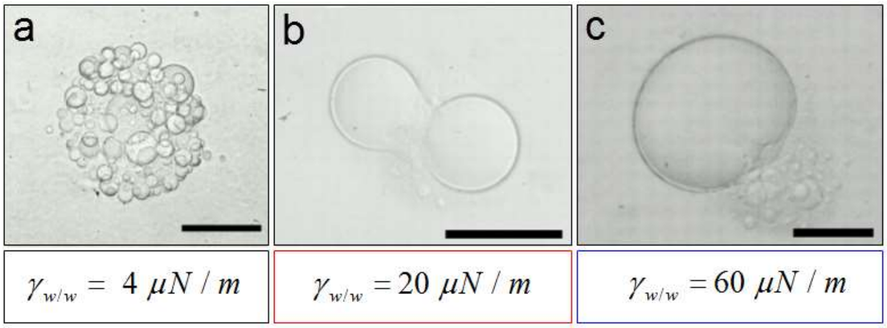

Supplementary Fig. 7 Effect of interfacial tension on the budding-like division of w/w droplets. The diameter of dividing droplets increases with increasing w/w interfacial tension of the water-in-water emulsion. The applied osmotic pressure is 25 mOsm kg^{-1}. The w/w interfacial tensions $\gamma_{w/w}$ are: (a) 4 μN m^{-1}; (b) 20 μN m^{-1}; and (c) 60 μN m^{-1}. The initial concentration of fibrils in the droplet is 1%. Scale bar is 200 μm.

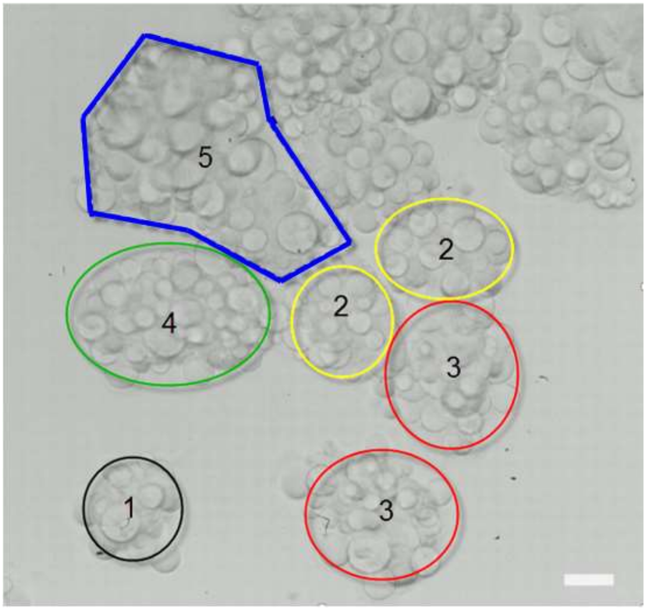

Supplementary Fig. 8 The final diameter of the stabilized buds is independent of the volume of the mother droplet. Optical microscope image of dividing mother droplets with different initial diameters. The boundary lines enclose daughter droplets originating from the same mother droplet, whose relative size is indicated by a number running from 1 to 5. A larger number represents a larger volume of the w/w droplet. The concentration of fibrils is 1.5 wt%. This figure illustrates that the diameter of the stabilized daughter droplets in independent of the volume of the original mother droplet. Scale bar is 100 μm.

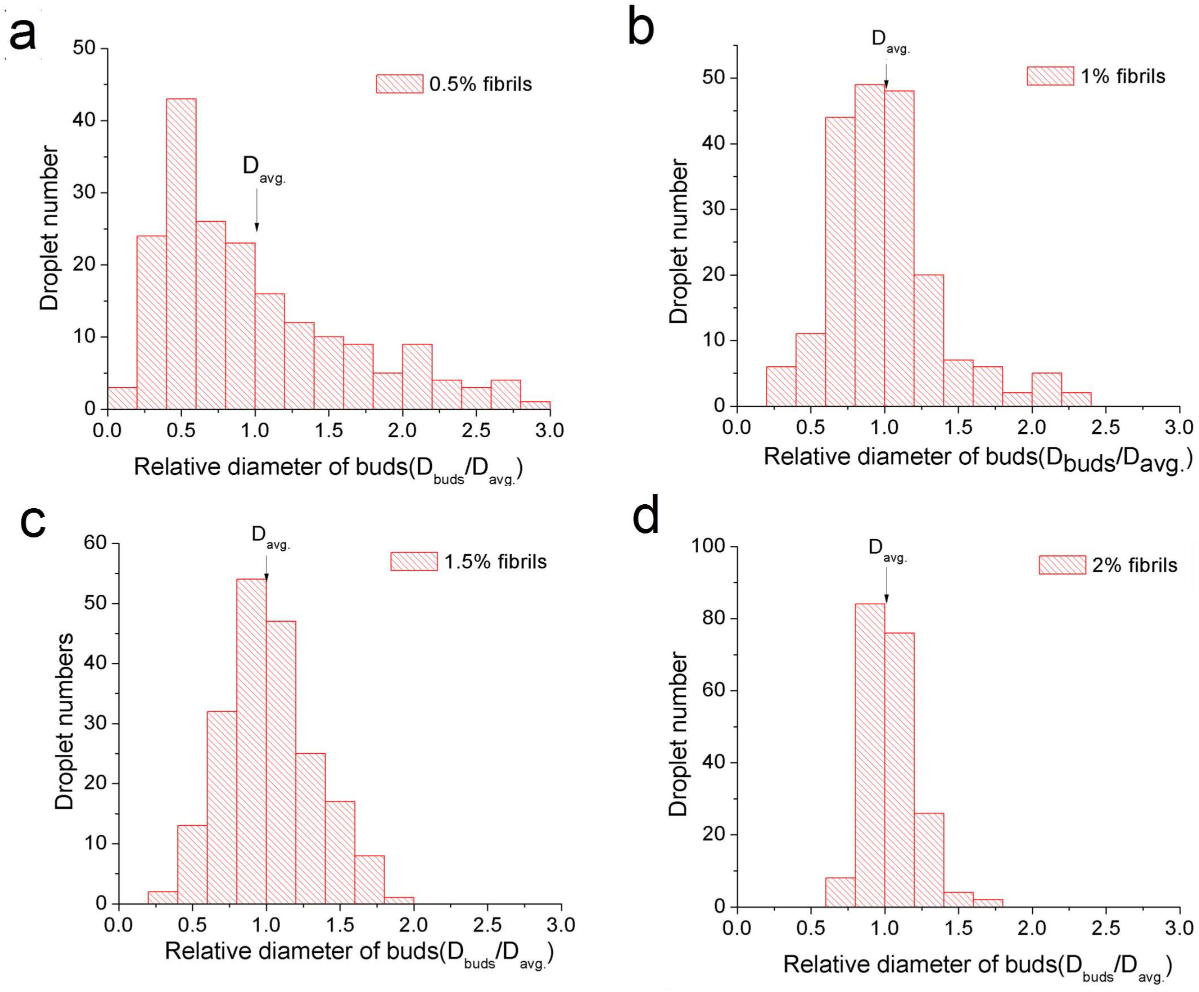

Supplementary Fig. 9 Size distribution of daughter droplets as a function of initial fibril concentration in the droplets. The concentration of dextran in the droplet phase was maintained at 4% and the PEG concentration in the continuous phase is 8%. For each concentration, 200 daughter droplets were measured. $D_{avg.}$ represents the average diameter of these daughter droplets, D_{buds} represent the diameter of each daughter droplet.

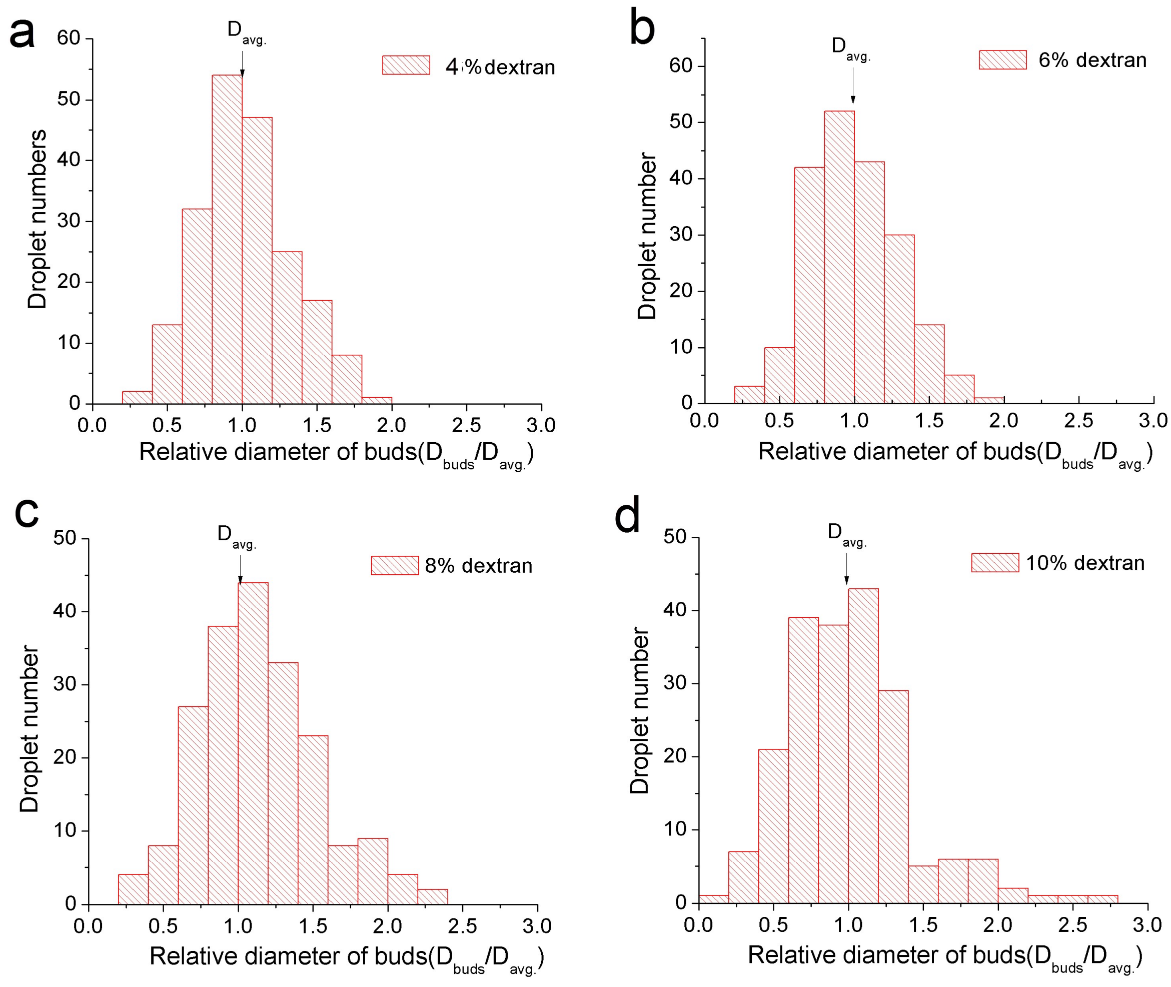

Supplementary Fig. 10 Size distribution of daughter droplets as a function of dextran concentration in the droplets. The initial fibril concentration in the droplet phase was maintained at 1.5 wt% and the PEG concentration in the continuous phase was 8%. For each concentration, 200 daughter droplets were measured. D_{avg}. represents the average diameter of these daughter buds, D_{buds} represent the diameter of each bud.

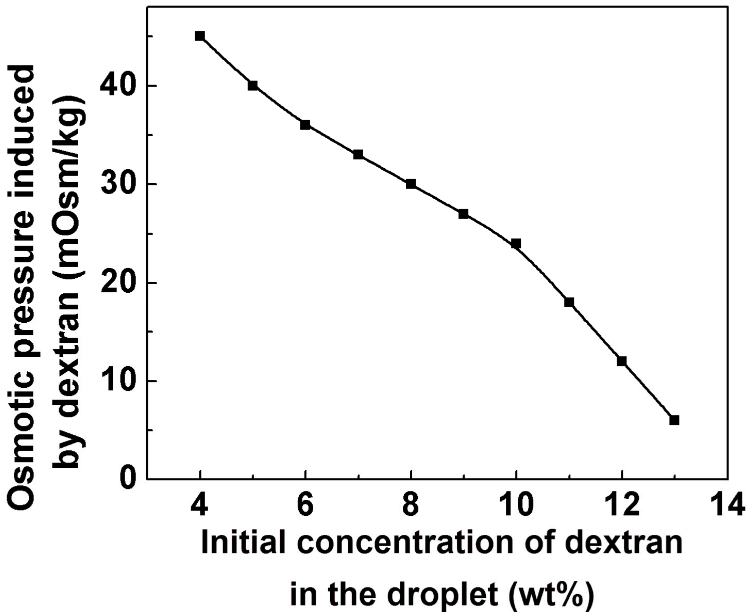

Supplementary Fig. 11 Change in the osmotic pressure induced by the condensation of the dextran-rich phase. When the initial concentration of dextran T500 in the droplet phase increases from 4 wt% to 13 wt%, the osmotic pressure between the droplet and continuous phase decreases. The concentration of PEG (Mw=20,000) in the continuous phase is maintained at 8 wt%.

Supplementary Tables

Supplementary Table 1. A list of experimental parameters for generating w/w emulsion with different interfacial tension under the same osmotic pressure (induced by dextran). The interfacial tension between the emulsion and continuous phases are measured using a spinning drop tensiometer. As the presence of fibrils does not significantly change the phase diagram of dextran-PEG-H_2O, the osmotic pressure induced by dextran is calculated by measuring the change in the osmolarity of dextran solution before and after budding without adding fibrils.

Group	Concentration of PEG 20,000 in continuous phase	Concentration of Dextran T500 in Phase Equilibrium	Concentration of Dextran before budding	Interfacial tension ($\mu N\ m^{-1}$)	Osmotic pressure ($mOsm\ kg^{-1}$)
A	6.7 wt%	10.9 wt%	4 wt%	4	25
B	7.6 wt%	13.6 wt%	8.4 wt%	20	25
C	10.4 wt%	16.5 wt%	11.5 wt%	60	25

Supplementary Notes

Supplementary Note 1-- Dewetting of fibril networks from the droplet phase

To enable the dewetting of the dextran-rich subdroplets (in red color) from the surface of the fibril networks (in green color), the various interfacial tensions (line tensions) in the system must meet the following condition.

$$\sigma_{\mathrm{w/w}} \cos\theta + \sigma_{\mathrm{dex/net}} > \sigma_{\mathrm{PEG/net}} \qquad (1)$$

Where the interfacial tension $\sigma_{\mathrm{dex/net}}$ and $\sigma_{\mathrm{w/w}}$ can be measured using a tensiometer, as shown in SI Fig.3b. Since the fibrils do not phase separate in the PEG-rich phase, so $\sigma_{\mathrm{PEG/net}} = 0\ \mu\mathrm{N/m}$.

The wetting angle, θ, (Supplementary Fig. 3) increases from 0° to 180° during the dewetting process.

Since $\sigma_{\mathrm{dex/PEG}} > \sigma_{\mathrm{dex/net}}$, in the force equilibrium state, we obtain a wetting angle of

$$\theta = \mathrm{acrcos}(-\frac{19}{33}) = 125^\circ \quad > 90^\circ \qquad (2)$$

which is in agreement with the observed wetting angle in Fig. 2f and Fig. 2g in the main text. The calculated wetting angle is also close to the contact angle measured directly by sitting a dextran-in-PEG droplet on a flat film of fibrils (142°), see supplementary reference 1.

When the fibril concentration is varied from 0.5 wt% to 2 wt%, the interfacial tension between the dextran and the fibril network $\sigma_{\mathrm{dex/network}}$ remains unchanged. We found that both $\sigma_{\mathrm{dex/network}}$ and

$\sigma_{\text{dex/PEG}}$ increase with the dextran concentration, and the $\sigma_{\text{dex/PEG}}$ is always higher than $\sigma_{\text{dex/network}}$ for all experimental conditions tested (dextran=5 wt%, 7.5 wt%, 10 wt%, 12.5 wt%, 15 wt%).

Supplementary Note 2 -- Estimation of the osmotic pressure prevailed in the budding-like division

In our experiment, water with 8% PEG and 10mM HCl is used as the continuous phase. The droplet phase is water with 4%-13% dextran and 1% fibrils. After suspending the droplet phase into a large volume of PEG-rich continuous phase, water is extracted from the droplet phase. Due to the resultant dehydration, the concentration of dextran in the droplet phase increases to about 15 wt% (46 mOsm kg^{-1}), as can be determined from the equilibrium phase diagram of dextran/PEG/H_2O (see Supplementary Reference 2). By measuring the osmolarity of the initial dextran-rich droplet phase, the change in the osmolarity in the droplet phase, induced by the condensation of dextran-rich phase, can be calculated.

We emphasize here that the fibril network does not contribute to the osmolarity of the droplet phase significantly, due to the limited number of fibrils relative to dextran molecules. Small molecules also do not contribute to the osmotic pressure, because they can migrate across the w/w interface. With these assumptions, the osmolarity induced by dextran in the droplet phase can be evaluated.

Supplementary Note 3 -- The size of stabilized daughter droplets is controlled by fibrils concentration, shrinkage ratio and w/w interfacial tension.

In this section, we present a simple model for understanding the expected dependence of the size of daughter droplets on the nanofibril concentration, as described in Equation (2) in the main text. We consider a 'mother' w/w emulsion droplet of volume V_{mother} (prior to dehydration) initially containing a concentration of fibrils, C_{fibril} (expressed in wt%). The volume of the mother droplet V_{mother} is related to the total volume of the daughter droplets V after dehydration through the shrinkage ratio η as:

$$\eta = \frac{V_{\text{mother}}}{V} \tag{3}$$

As the dextran-rich phase is a bad solvent for fibrils, fibrils form bundles in the dextran-rich phase. Assuming these fibril bundles have cylindrical shape with cross-sectional radius a and length l, the volume of each fibril bundle is $V_{\text{fibrils}} = \pi w^2 l$; hence, the total number of bundles in the w/w droplet, N, can be estimated as

$$N_{\text{fibril}} = \frac{m_{\text{fibrils}}}{m_{\text{single fibril}}} = \frac{m_{\text{mother}} C_{\text{fibril}}}{\rho_{\text{fibril}} V_{\text{bundle}}} = \frac{\rho_{\text{mother}} V_{\text{mother}} C_{\text{fibril}}}{\rho_{\text{fibril}} \pi a^2 l} = \frac{\rho_{\text{mother}} \eta V C_{\text{fibril}}}{\rho_{\text{fibril}} \pi a^2 l} \tag{4}$$

where m_{mother} & ρ_{mother} are the mass and density of the mother droplet, respectively;

C_{fibril} & ρ_{fibril} are the initial concentration and density of fibrils in the mother droplet, respectively;

using $\rho_{\text{mother}} \approx \rho_{\text{fibril}}$ =1.0 mg cm^{-3}, we arrive at:

$$N_{\text{fibril}} = \frac{\eta V C_{\text{fibril}}}{\pi a^2 l} \tag{5}$$

We now consider the splitting of the w/w emulsion droplet (volume V after dehydration) into several daughter droplets with average radius R. The number of daughter droplets N_d that result from this division is:

$$N_d = \frac{3V}{4\pi R^3} \tag{6}$$

Hence, the surface area of the w/w interface after division is given by:

$$A_d = 4\pi R^2 N_d = \frac{3V}{R} \tag{7}$$

To stabilize the w/w interface, the fibril bundles dewet from the dextran-rich phase and adsorb onto the w/w interface of daughter droplets. In this process, the adsorption energy per fibril bundle is:

$$\Delta G = \sigma_{w/w} al[\sin(\pi - \theta) + \theta \cos(\pi - \theta)] \approx \sigma_{w/w} \pi al \cos\theta \tag{8}$$

(see Supplementary Reference 1, Supplementary Note 4 for the deduction of the above adsorption energy).

Note that the approximation used in the last step is valid for large contact angles (here θ=142°).

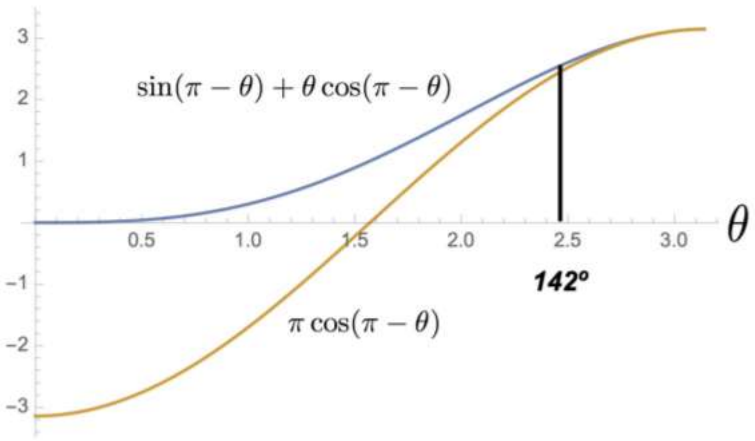

The contact angle θ is related to the surface tension $\sigma_{\mathrm{w/w}}$ of the w/w interface as well as the surface tensions $\sigma_{\mathrm{dex/net}}$ and $\sigma_{\mathrm{PEG/net}}$ between the fibrils and the two aqueous phases through the Young-Dupré equation:

$$\cos\theta = \frac{\sigma_{\mathrm{PEG/net}} - \sigma_{\mathrm{dex/net}}}{\sigma_{\mathrm{w/w}}} \tag{9}$$

Assuming all fibril bundles migrate to the w/w interface and stabilize the daughter droplets, (w/w surface tension) × (total surface area of stabilized w/w interface) = (total number of fibril bundles) × (adsorption energy per fibril), combing Supplementary Equation (5), (7), (8) and (9), we obtain

$$\sigma_{\mathrm{w/w}} \times \frac{3V}{R} = \frac{\eta V C_{\mathrm{fibril}}}{\pi a^2 l} \times \sigma_{\mathrm{w/w}} \pi a l \left|\cos\theta\right| = \frac{\eta V C_{\mathrm{fibril}}}{l} (\sigma_{\mathrm{dex/net}} - \sigma_{\mathrm{PEG/net}}) \tag{10}$$

where we also assume the coverage ratio of fibril bundles at the w/w interface, $\phi : 1$.

Solving this equation for the diameter of stabilized daughter droplets, we find

$$D = 2R = \frac{3a\sigma_{\mathrm{w/w}}}{(\sigma_{\mathrm{dex/net}} - \sigma_{\mathrm{PEG/net}})\eta C_{\mathrm{fibril}}} \tag{11}$$

Note that, according to Supplementary Equation (11), the average diameter of daughter droplets is independent of the volume of the mother droplet V_{mother}, in accordance with what is observed in Supplementary Fig. 8. Moreover, D is inversely proportional to the combined product of the concentration of fibrils, C_{fibril}, and the shrinkage ratio of the mother droplet, η (see Fig. 3 in the main text).

An example for mathematic prediction of the size of the daughter droplets.

When both the concentration of dextran and PEG in the droplet and the continuous phases are 7.5 wt%, the corresponding shrinkage ratio of the droplet

$$\eta = \frac{c(\text{dextran})_{\text{after dehydration}}}{c(\text{dextran})_{\text{before dehydration}}} = \frac{15.2\%}{7.5\%} = 2.0 \tag{12}$$

the initial concentration of fibrils in the droplet phase is 1.2%; the w/w interfacial tension, as well as the interfacial tension between the dextran-rich phase and the network can be measured from the tensiometer, $\sigma_{\text{w/w}} = 33 \mu N\, m^{-1}$ and $\sigma_{\text{dex/net}} = 19 \mu N\, m^{-1}$; therefore,

$$D_{\text{daughter}} = 2R_{\text{daughter}} = \frac{3 w_{\text{bundle}} \sigma_{\text{w/w}}}{2\sigma_{\text{dex/net}} \eta C_{\text{fibril}} f(\theta)} = \frac{3 \times 1.5\mu\text{m} \times 33\mu\text{N m}^{-1}}{2 \times 19\mu\text{N m}^{-1} \times 2 \times 1.2\% \times 0.8} = 200\mu\text{m} \tag{13}$$

Which is approximately equal to the measured diameter of the daughter droplets, 170~180 μm (see Fig.1a in the main text).

Supplementary Note 4 -- Effect of interfacial tensions on the budding droplet morphology

The w/w interfacial tension contributes to droplet division since it sets the magnitude of the resistance against formation of surface protrusions and the maximum curvature of the resultant protrusions. To demonstrate this, we increase the interfacial tension $\gamma_{w/w}$ from 4 μN m^{-1} to 60 μN m^{-1} by increasing the PEG concentration in the continuous phase; meanwhile, we also increase the dextran concentration in the droplets to keep the same shrinkage ratio and the actual fibril concentration (see Supplementary Table 1). As the interfacial tension increases, merging of the protrusions was observed to occur more frequently, resulting in protrusions with increased diameters. When the interfacial tension is higher than 60 μN m^{-1}, nucleation of the protrusion is constrained or even inhibited. When interfacial tension is less than 4 μN m^{-1}, osmotic flow can cause fluctuations of the w/w interface and the uncontrolled breakup of the mother droplet.

Supplementary Note 5 --- Effect of fibril concentration on formation of new interfacial area.

The increased interfacial area of the daughter droplets after division,

$$\sum_n S_{\text{daughter}} = \frac{3V}{R_{\text{daughter}}} = \frac{4\sigma_{\text{dex/net}} C_{\text{fibril}} \eta}{w_{\text{bundle}} \sigma_{\text{w/w}}} g \frac{V_{\text{mother}}}{\eta} = \frac{4 \times 19 \times C_{\text{fibril}}}{1.5 \times 10^{-6} \times 33} \times \frac{4\pi}{3} \times (\frac{250}{2} \times 10^{-6})^3 = 1.26 \times 10^{-5} C_{\text{fibril}} \quad (14)$$

Since the width of bundles, w_{bundle}, does not change with the concentration of fibrils, so the total surface area of daughter droplets should increase linearly with the concentration of fibrils.

For a w/w droplet without addition of fibrils, the surface area of this fibril-free droplet after dehydration is

$$S_{\text{dehydration}} = 4\pi R_{\text{dehydration}}^2 = 4\pi(\frac{1}{\eta})^{\frac{2}{3}} R_{\text{mother}}^2 = 4\pi(\frac{1}{2})^{\frac{2}{3}}(\frac{2.5\times10^{-4}}{2})^2 = 1.24\times10^{-7}\ \text{m}^3 \tag{15}$$

The ratio of the surface area of the daughter droplets relative to that of the fibril-free droplet after dehydration is estimated from the following equation,

$$\frac{\sum_n S_{\text{daughter}}}{S_{\text{dehydration}}} = \frac{1.26\times10^{-5} C_{\text{fibril}}}{1.24\times10^{-7}} = 102 C_{\text{fibril}} \tag{16}$$

as shown by the estimated blue plot in Fig. 3b of the main text.

Supplementary Note 6 -- Effect of shrinkage ratio on the number and size of daughter droplets.

Dehydration condenses the fibril network in the droplet and thus increases the actual concentration of fibrils. We define a combined parameter,

$$X = \eta C_{\text{fibril}} \tag{17}$$

where X represents the concentration of fibrils in the droplet after dehydration.

$$R = \frac{3\sigma_{\text{w/w}} a}{2\sigma_{\text{dex/network}} \eta C_{\text{fibril}}} = \frac{3\times33\times1.5\times10^{-6}}{2\times2\times19\times0.8} \bullet \frac{1}{X} = \frac{2.44\times10^{-6}}{X}\ (m) \tag{18}$$

For multiple division, the number of daughter droplets $n \geq 2$; For single division, the number of daughter droplets $1 < n < 2$; The boundary line separating the single and multiple division can be estimated by

$$n = \frac{3V}{4\pi R^3} = 2 \tag{19}$$

Since

$$R = \frac{3\sigma_{\mathrm{w/w}} a}{2\sigma_{\mathrm{dex/net}} \eta C_{\mathrm{fibril}}} \tag{20}$$

Hence,

$$\eta^2 C_{fibrils}^3 = \frac{9\pi \sigma^3_{\mathrm{w/w}} a^3_{\mathrm{bundle}}}{V_{\mathrm{mother}} \sigma^3_{\mathrm{dex/net}}} = \frac{9\pi \times (33 \times 1.5 \times 10^{-6})^3}{8 \times \frac{4\pi}{3} (1.25 \times 10^{-4})^3 \times 19^3} = 7.6 \times 10^{-6} \tag{21}$$

Plot the critical concentration of fibrils $C^*_{fibrils}$ against the shrinkage ratio of the w/w emulsions results in the estimated phase diagram in Fig.3e of the main text.

Supplementary References

(1) Song, Y. et al. Fabrication of fibrillosomes from droplets stabilized by protein nanofibrils at all-aqueous interfaces. *Nat. Commun.* 7, 12934 (2016).

(2) Nguyen, B. T., Nicolai, T. & Benyahia, L. Stabilization of water-in-water emulsions by addition of protein particles. *Langmuir* **29**, 10658-10664 (2013).
